# Magnetic Resonance Imaging of Endolymphatic Hydrops Using Compressed Sensing Acceleration Technique

**DOI:** 10.7759/cureus.91932

**Published:** 2025-09-09

**Authors:** Yukiyoshi Kimura, Ricardo Ceballos-Lizarraga, Dafne Soto-Trujillo, Cesar N Cristancho-Rojas

**Affiliations:** 1 Radiology, Instituto Nacional De Ciencias Medicas y Nutricion Salvador Zubiran, Mexico City, MEX; 2 Neurology, Clinica de Vertigo y Mareo Santa Fe, Mexico City, MEX; 3 Neurology, The Vestibular Project Initiative, Mexico City, MEX; 4 Radiology, Centro Medico ABC, Mexico City, MEX; 5 Radiology, CT Scanner Mexico, Mexico City, MEX

**Keywords:** endolymphatic hydrops, hydrops, magnetic resonance (mr), meniere’s disease, peripheral vertigo

## Abstract

Endolymphatic hydrops (EH) is a key underlying pathologic alteration of Ménière's disease (MD). MD is characterized by recurrent episodes of spontaneous vertigo, fluctuating sensorineural hearing loss, tinnitus, and aural fullness. Magnetic resonance imaging (MRI) plays a crucial role in visualizing EH, with gadolinium-based contrast agents facilitating the identification of endolymph and perilymph. Specific MRI techniques, such as the hybrid of reversed image of positive endolymph signal and native image of positive perilymph signal (HYDROPS) imaging method, offer high-quality results but are limited by long acquisition times. This study explores the use of compressed sensing to accelerate image acquisition and improve contrast-to-noise ratio (CNR) in EH imaging in a 3 Tesla magnet using a 16-channel array head coil, comparing a new proposed cs-HYDROPS sequence with the conventional HYDROPS technique. A total of 24 ears from 12 patients with suspected MD were analyzed using both methods. The cs-HYDROPS sequence demonstrated a substantial reduction in scan time with a significant reduction in acquisition time of 57.3% (from 30.2 minutes to 12.9 minutes) while achieving a 143.5% increase in CNR (mean CNR for cs-HYDROPS: 103.8; SD: 37.3). Diagnostic agreement between the two methods had a Cohen's kappa value of 0.903. These findings demonstrate that cs-HYDROPS offers a reliable and efficient alternative for evaluating EH in MD patients, reducing scan time while enhancing image quality and maintaining diagnostic accuracy. This technique may facilitate wider clinical adoption of EH imaging in the routine workup of MD.

## Introduction

Ménière's disease (MD) is an inner ear disorder characterized by recurrent episodes of spontaneous vertigo, fluctuating sensorineural hearing loss, tinnitus, and aural fullness [1-3]. The key underlying pathologic alteration is endolymphatic hydrops (EH), as demonstrated by both histopathological and magnetic resonance imaging (MRI) studies. Symptoms and the underlying EH typically begin in one ear, although progression to bilateral involvement can occur over time. 

The age of clinical onset for MD exhibits a wide distribution, from childhood through older age. However, most individuals experience clinical onset in adulthood, with peak incidence observed between 40 and 60 years. Estimates indicate that 50 to 200 per 100,000 individuals are affected by this disorder [3]. MD is characterized by a chronic and progressive clinical course, resulting in the gradual loss of vestibular and auditory functions. Reported incidence rates range from 4.3 per 100,000 per year in Finland to 13.1 per 100,000 person-years in the UK [4-5].

MRI for the detection of EH was initially performed using intratympanic administration of gadolinium-based contrast agents (GBCA). This technique employed three-dimensional fluid-attenuated inversion recovery (3D-FLAIR) sequences on a 3 Tesla system, utilizing low GBCA volumes to achieve a high contrast-to-noise ratio (CNR). However, this semi-invasive procedure entails its off-label use [6]. Currently, MRI evaluation of EH via intravenous administration of GBCAs represents a noninvasive and clinically accessible technique for diagnosing atypical MD and bilateral subclinical disease [7]. This technique has been primarily utilized in clinical research.

Development of positive perilymph and endolymph images acquired using heavily T2-weighted 3D-FLAIR sequences with a constant flip angle and readout echo train, four hours post-GBCA intravenous administration, enables the visualization of both fluids as a bright signal. Hybrid of reversed image of positive endolymph signal and native image of positive perilymph signal (HYDROPS) images are the resulting subtraction and have been employed for several years to diagnose EH in clinical practice [8].

Furthermore, modest reductions in conventional scan times have been achieved. An initial total scan time of 30 minutes, comprising 15 minutes per sequence, has been reduced by utilizing improved HYDROPS imaging, referred to as iHYDROPS6. iHYDROPS images can be acquired in half the time, with a total acquisition time of 14 minutes, while achieving a higher CNR by increasing the flip angle and inversion times [9]. This is accomplished using a 3 Tesla scanner equipped with a 32-channel array head coil. Acquisition times can be further reduced, and CNR improved, by employing newer acceleration techniques such as compressed sensing, in conjunction with smaller slice thickness images and fewer-channel array head coils. In our study, the resulting image was termed as cs-HYDROPS image. A higher CNR between the perilymph and endolymph improves visualization of the diminutive endolymphatic structures within the membranous labyrinth, including the saccule and utricle in the vestibule, and the scala media in the cochlea.

Despite advances in MRI techniques, the main drawback of these acquisition methods remains the prolonged acquisition time required for positive endolymph and perilymph sequences. Our study's primary aim was to compare the CNR between positive endolymph/positive perilymph images and HYDROPS images when using a new cs-acquisition technique. Our goal was to reduce acquisition times while maintaining CNR. Additionally, we examined whether there was a significant difference in diagnostic performance between traditional HYDROPS images and cs-HYDROPS images.

## Materials and methods

This cross-sectional study was conducted at a reference private imaging center in Mexico City. Participant recruitment employed convenience sampling, selecting individuals presenting with clinical indications for inner ear MRI and meeting criteria for probable or definite MD according to the 2015 American Academy of Otolaryngology-Head and Neck Surgery (AAO-HNS) guidelines [10]. Diagnosis and classification were established by an otoneurologist who determined the clinical necessity for MRI referral. Subsequently, all participants underwent an MRI to evaluate EH. The cs-HYDROPS scan was added to the conventional protocol. All participants had an estimated glomerular filtration rate greater than 60 ml/min/1.73 m^2^. This study received approval from the institution's ethics committee, and informed consent was obtained from all participants.

MRI

All MRI was performed using a 3 Tesla scanner (Lumina, Siemens, Erlangen, Germany) with a 16-channel array head coil. MR scanning was performed four hours after IV-GBCA (0.2 ml/kg body weight or 0.1 mmol/kg) of gadobutrol (Gadovist, Bayer Schering Pharma, Leverkusen, Germany).

According to the conventional protocol for the evaluation of EH, patients underwent a 0.3 mm Sampling Perfection with Application optimized Contrast using different flip angle Evolution (T2-SPACE) sequence as an anatomical reference of the total lymph fluid, a 3D-SPACE-T2-FLAIR scan with a GeneRalized Autocalibrating Partial Parallel Acquisition (GRAPPA) acceleration factor of 2, a slice thickness of 1.0 mm, a 9000 msec TR, a 2250 msec inversion time, and a slice oversampling of 50% for the positive perilymph image. A positive endolymph image was acquired with the same parameters and a 2050 msec inversion time at four hours after the administration of the IV-SD-GBCA. The maximum refocusing flip angle in the echo train was 130 degrees for both images. The total scan time of the HYDROPS image was 30.2 minutes.

The cs-HYDROPS image with a shorter acquisition time was obtained after the conventional protocol scan (270 minutes after GBCA administration); the same TR, maximum refocusing flip angle, time to inversion, and slice oversampling were used as in the conventional acquisition, with a thinner slice thickness of 0.7 mm and a compressed sensing acceleration factor of 6. The total scan time of the cs-HYDROPS image was 12.9 minutes. The number of excitations was reduced from four to 3.4. Detailed scan parameters are shown in Table 1.

**Table 1:** Technical parameter of MRI sequence acquisition MRI: magnetic resonance imaging; RT: repetition time; ET: echo time; IT: inversion time; PPI: positive perilymph image; PEI: positive endolymph image; CS: compressed sensing; GRAPPA: GeneRalized Autocalibrating Partial Parallel Acquisition

Sequence name	Type	RT (ms)	ET (ms)	IT (ms)	Flip angle (degree)	Section thickness (mm)	Voxel size	Number of excitations	Acceleration technique	Acceleration factor	Scan time (mins)
Conventional PPI	3D Space	9000	543	2250	130	1	0.4 x 0.4	4	GRAPPA	2	15.11
Conventional PEI	3D Space	9000	543	2050	130	1	0.4 x 0.4	4	GRAPPA	2	15.11
CS PPI	3D Space	9000	543	2250	130	0.7	0.4 x 0.4	3.4	Compressed sensing	6	6.47
CS PEI	3D Space	9000	543	2050	130	0.7	0.4 x 0.4	3.4	Compressed sensing	6	6.47

Image processing and analysis

The image dataset was transferred to an imaging workstation (syngo.via Siemens, Erlangen, Germany). The HYDROPS and cs-HYDROPS images were generated by subtracting the positive endolymph image from the positive perilymph image acquired with the conventional and new protocols, respectively. A neuroradiologist with five years of experience performed the image analysis on an imaging workstation. Each of the 24 studied ears was blindly classified as positive or negative for EH. The CNR between the perilymph and endolymph was measured using conventional HYDROPS and cs-HYDROPS images in each of the ears per patient, resulting in two measures of CNR per scan.

**Figure 1:**
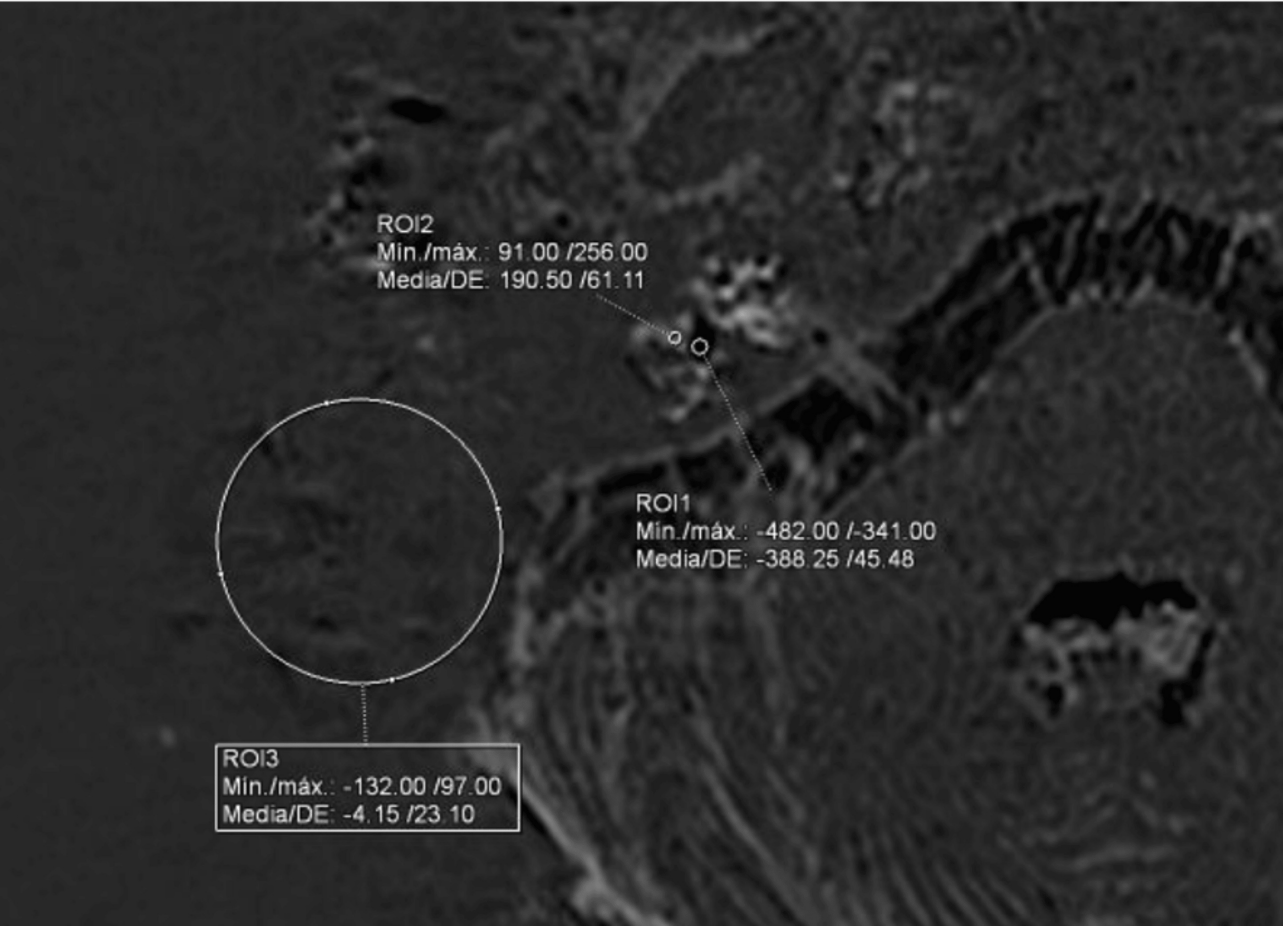
Region-of-interest placement ROI: region of interest; CNR: contrast-to-noise ratio; HYDROPS: hybrid of reversed image of positive endolymph signal and native image of positive perilymph signal An example of an ROI placement on a cs-HYDROPS image. ROI 1 was placed in the vestibular endolymph at the utricle, as large as possible. ROI 2 was drawn in the perilymph of the vestibule. A 20 mm circular ROI 3 was placed on the lateral side of the labyrinth in a uniform signal intensity area for the noise evaluation. The CNR was evaluated as follows: CNR = (signal intensity of perilymph ROI-signal intensity of endolymph ROI)/standard deviation of noise ROI

A circular 20 mm diameter region of interest (ROI) was placed on the lateral side of the labyrinth in a uniform signal intensity area for noise evaluation. For the endolymph evaluation, a circular ROI was placed on the utricle at the vestibule, and a circular ROI was drawn on the scala tympani at the cochlear basal turn or the perilymph of the vestibule. Details of the ROI placement were described previously by Naganawa et al. [11]. An ROI placement example is shown in Figure 1. For the HYDROPS and cs-HYDROPS images, the CNR was defined as the subtraction of the signal intensity value of the perilymph ROI minus the signal intensity value of the endolymph ROI divided by the standard deviation of the noise ROI. High intraclass correlation coefficients between the endolymphatic area ratios by two different observers have been reported [12]. Signal intensities of the left and right ears were averaged for each patient.

Sample size calculation

The sample size was determined based on prior research comparing inner ear MRI HYDROPS sequences and utilizing an online sample size calculator [12-13]. We calculated the sample size needed to detect a minimum difference of 40%, assuming a CNR mean of 90.3 and a standard deviation of 29.2. Using a two-tailed t-statistic (with a noncentrality parameter) to compare means, we determined that 12 observations per group were required.

Statistical analysis

Quantitative variables were summarized using central tendency and dispersion measures, specifically the mean with SD and the median with interquartile range (defined as p25-p75). Categorical variables were expressed as percentages. The normality of CNR data was assessed using the Shapiro-Wilk test, and the homogeneity of variance among sequence measurements was evaluated with Levene’s test. The agreement between EH diagnosis and conventional HYDROPS sequences, based on independent and blinded radiologist interpretation, was assessed using a Cohen's kappa test.

Furthermore, the intraclass correlation coefficient assessed the consistency between right and left CNR values within the same sequence acquisition. The average of the CNRs from both ears was calculated to determine a global CNR for each scan. These global CNRs were utilized to compare conventional with cs-HYDROPS sequences, performing either an independent t-test or the Mann-Whitney U test in accordance with the results of the normality assessment. All statistical analyses were performed using R version 4.4.2 (Copyright 2024 The R Foundation for Statistical Computing Platform), and a p-value of <0.05 was considered statistically significant.

## Results

A total of 24 ears of 12 patients with clinical suspicion of EH were included in the study. The sample consisted of seven women (58.3%), with ages ranging from 25 to 71 years (mean: 46.8 years; SD: 12.9). Demographic details are shown in Table 2. For the radiologic diagnostic assessment of EH, blind and independent evaluations resulted in only one discordant result: one ear was classified as positive in the cs-HYDROPS sequence but negative in the conventional sequence. A statistically significant agreement was reached in the diagnosis of EH across the two image sequences' independent evaluations, evidenced by a kappa coefficient of 0.903.

**Table 2:** Demographic data of patients Values are presented as mean (standard deviation) for continuous variables and percentages for categorical variables

Demographic characteristics	
Age mean (SD)	46.8 years (12.96)
Sex (female %)	58.30%

Table 3 summarizes the central tendency and dispersion statistics for CNR measurements in each ear. The distribution of CNR values for both sequences did not significantly deviate from normality (Shapiro-Wilk test, p > 0.05). Additionally, high consistency was observed in CNR values between the right and left ears within the same sequence acquisition, supported by an intraclass correlation coefficient of 0.99.

**Table 3:** Central tendency and dispersion statistics for CNR measurements CNR: contrast-to-noise ratio; SD: standard deviation; IQR: interquartile range; ICC: intraclass correlation coefficient; HYDROPS: hybrid of reversed image of positive endolymph signal and native image of positive perilymph signal The cs-HYDROPS sequence showed a significantly higher mean global CNR (103.8 ± 37.3) than the conventional sequence (42.6 ± 16.8), with a mean difference of 61.2 (143.5% increase; two-sample t-test with p-value < 0.001). Distributions did not deviate from normality (Shapiro-Wilk, p > 0.05), and inter-ear consistency was excellent (ICC = 0.99). No heteroscedasticity was detected (Levene’s test, p = 0.17)

Variable	Mean (SD)	Median (IQR)
cs-HYDROPS sequence		
Right ear	103.1 (37.3.)	95.6 (82.0, 121.0)
Left ear	104.4 (38.2)	97.2 (82.9, 121.8)
Global	103.8 (37.3)	96.37 (39.15)
Conventional HYDROPS sequence		
Right ear	42.7 (16.8)	40.1 (29.8, 50.2)
Left ear	42.5 (17.1)	37.8 (29.9, 51.4)
Global	42.6 (16.8)	38.94 (21.45)
CNR global difference cs-HYDROPS vs. conventional	143.5% (61.2)	t-test p-value < 0.001

**Figure 2:**
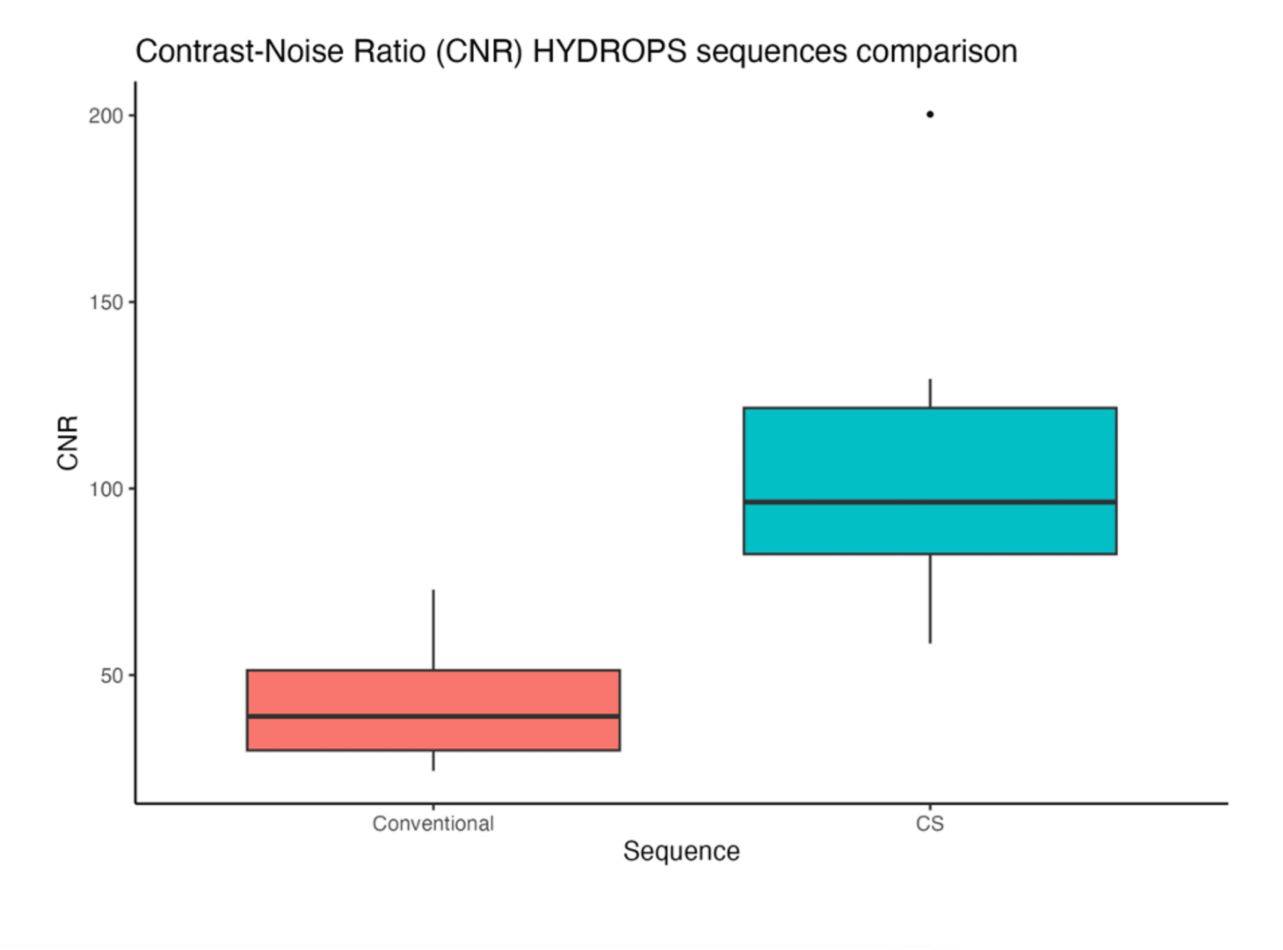
CNR HYDROPS sequences comparison CNR: contrast-to-noise ratio; SD: standard deviation; HYDROPS: hybrid of reversed image of positive endolymph signal and native image of positive perilymph signal Box plot of mean and SD illustrating the CNR distribution for cs-HYDROPS and conventional sequences

The cs-HYDROPS sequence demonstrated a significantly higher mean global CNR (103.8, SD: 37.3) compared to the conventional sequence (42.7, SD: 16.8). No evidence of heteroscedasticity was observed between the two groups (Levene’s test, p = 0.17). The cs-HYDROPS sequence exhibited a mean CNR difference of 61.2, corresponding to a 143.5% increase over the conventional sequence shown in Figure 2.

## Discussion

Our study demonstrated that the cs-HYDROPS sequence reduced MRI acquisition time by over 62% while simultaneously achieving a 143.5% increase in the mean global CNR compared to the conventional HYDROPS sequence. Moreover, excellent agreement was observed in the diagnostic impression of EH (Cohen's kappa = 0.903). CNR values for both sequences also exhibited remarkable consistency between the right and left ears (ICC = 0.99). MRI of EH is anticipated to play a pivotal role in the evaluation and workup of MD [14]. Consequently, enhancing image quality remains essential for its broader clinical application. Our findings underscore the robustness and reliability of the cs-HYDROPS sequence for the evaluation of EH.

The compressed sensing technique, as employed in the HYDROPS MRI sequence in our study, is a signal processing approach that enables the reconstruction of signals from a reduced number of measurements [15]. This methodology has been effectively utilized to accelerate the acquisition process in MRI [16]. Key components of compressed sensing include signal sparsity, pseudorandom undersampling, and nonlinear iterative reconstruction methods [17]. This technique offers several benefits, such as the capacity to employ higher acceleration factors while maintaining and even enhancing image quality. Furthermore, it facilitates decreased acquisition times and enhances CNR, thereby enabling imaging with thinner slice thicknesses. In our study, a slice thickness of 0.7 mm in the cs- HYDROPS images led to the identification of EH in one ear, which had been classified as negative in the conventional HYDROPS images.

Numerous investigations have implemented HYDROPS imaging utilizing high-field strength magnets, often in conjunction with 32-channel array head coils, which can limit broader clinical adoption to specialized centers [18-20]. To the best of our knowledge, the present study constitutes the initial report of clinically reliable, high-resolution EH imaging achieved with a 16-channel array head coil and intravenous GBCA administration at 3 Tesla. Furthermore, Gurkov et al. developed a robust technique for EH grading at 1.5 Tesla (1.5T) using a 16-channel array head coil and a nine-minute acquisition time [21]. However, this methodology necessitates invasive contrast administration via intratympanic injection, which is an off-label procedure and restricts the ability to evaluate the contralateral ear. While the feasibility of HYDROPS imaging following intravenous administration of a single dose of GBCA at 1.5T has been previously documented utilizing a 32-channel array head coil, it is not yet widely adopted in routine clinical practice [22].

This study has several limitations. The MRI investigation of EH lacks a definitive gold standard based on pathological specimens. Additionally, there is a potential for case selection bias for convenience sampling. However, these two factors are similar in studies evaluating technical aspects of medical imaging. Each patient underwent four acquisitions, which could be prone to misalignment. Furthermore, the CS-positive perilymphatic and positive endolymphatic images were obtained after the conventional imaging, with more than 30 minutes having passed since the intravenous contrast injection. This delay may have affected the CNR. Future studies with larger sample sizes are recommended to address these issues, allowing for further clinical and pathological correlation.

## Conclusions

In this study, we compared conventional HYDROPS images with new cs-HYDROPS images. We observed a 143.5% increase in CNR in cs-HYDROPS images compared to the conventional sequence, with a significant reduction in acquisition time of 57.3% using a 16-channel array head coil. These benefits in acquisition time and enhanced image quality, while maintaining agreement in clinical diagnostic assessment, strongly suggest the potential for widespread adoption of this sequence in the clinical evaluation of MD.
